# On the Interaction of Head and Gaze Control With Acoustic Beam Width of a Simulated Beamformer in a Two-Talker Scenario

**DOI:** 10.1177/2331216519876795

**Published:** 2019-09-23

**Authors:** Ĺuboš Hládek, Bernd Porr, Graham Naylor, Thomas Lunner, W. Owen Brimijoin

**Affiliations:** 1 Hearing Sciences (Scottish Section), Division of Clinical Neurosciences, School of Medicine, University of Nottingham, UK; 2 School of Engineering, University of Glasgow, UK; 3 Eriksholm Research Centre, Oticon, Snekkersten, Denmark

**Keywords:** hearing aids, speech intelligibility, head movements, eye movements

## Abstract

Superdirectional acoustic beamforming technology provides a high signal-to-noise ratio, but potential speech intelligibility benefits to hearing aid users are limited by the way the users move their heads. Steering the beamformer using eye gaze instead of head orientation could mitigate this problem. This study investigated the intelligibility of target speech with a dynamically changing direction when heard through gaze-controlled (GAZE) or head-controlled (HEAD) superdirectional simulated beamformers. The beamformer provided frequency-independent noise attenuation of either 8 dB (WIDE [moderately directional]) or 12 dB (NARROW [highly directional]) relative to no beamformer referred as the OMNI (omni-directional) condition. Before the main experiment, signal-to-noise ratios were normalized for each participant and each beam width condition to yield equal percentage of correct performance in a reference condition. Hence, results are presented as normalized speech intelligibility (NSI). In an ongoing presentation, the participants (*n* = 17), of varying degree of hearing loss, heard single-word targets every 1.5 s coming from either left (−30°) or right (+30°) presented in continuous, spatially distributed, speech-shaped noise. When the target was static, NSI was better in the GAZE than in the HEAD condition, but only when the beam was NARROW. When the target switched location without warning, NSI performance dropped. In this case, the WIDE HEAD condition provided the best average NSI performance, because some participants tended to orient their head in between the targets, allowing them to hear out the target regardless of location. The difference in NSI between GAZE and HEAD conditions for individual participants was related to the observed head-orientation strategy, which varied widely across participants.

## Introduction

Attending a work meeting or having a conversation in a noisy cafeteria or restaurant is challenging for hearing-impaired people (Lebo et al., 1994). Hearing aids with forward-pointing directional microphones or microphone array beamformers attenuate the off-axis background noise and thus provide a signal-to-noise ratio (SNR) benefit for the signals directly in front of the listener (Bentler, 2005; Dillon, 2012). However, the benefit is limited in natural environments (Cord, Surr, Walden, & Dyrlund, 2004), and possible reasons include that people orient their heads toward the respective sound source in only about 30% to 50% of the listening time (Ching et al., 2009; Ricketts & Galster, 2008; Ricketts & Picou, 2010) and that directional microphones can make sounds more difficult to localize (Archer-Boyd, Holman, & Brimijoin, 2018; Brimijoin, Whitmer, McShefferty, & Akeroyd, 2014). For people wearing hearing aids with highly directional forward-pointing beamforming arrays, these problems might be even more pronounced (Best, Mejia, Freeston, van Hoesel, & Dillon, 2015; Buechner, Dyballa, Hehrmann, Fredelake, & Lenarz, 2014). Despite the myriad possible configurations of real-life situations, in social situations, like work meetings and conversations around a table in the cafeteria, target sources are most likely to be in the frontal hemisphere of the listener. Nevertheless, talkers are in different orientations, and a listener wearing hearing aids with highly directional microphones may miss the initial part of the utterance of a new talker from an unexpected direction.

Steering the directivity pattern of directional microphones with the eye gaze of the listener might be a possible solution to preserve the SNR benefits of beamforming arrays (Best, Roverud, Streeter, Mason, & Kidd, 2017; Favre-Félix, Graversen, Hietkamp, Dau, & Lunner, 2018; Hart, Onceanu, Sohn, Wightman, & Vertegaal, 2009; Roverud, Best, Mason, Streeter, & Kidd, 2018). In a preliminary study (Hart et al., 2009), gaze-steered target selection was compared with selection by physical pointing or button-pressing. The orientation of the target talker changed unpredictably every 10 s. The gaze-steered condition provided the fastest switching time and the best message recall. Despite that, in another study (Best, Streeter, Roverud, Mason, & Kidd, 2016) which used a Question-and-Answer paradigm, with target either “fixed” or “dynamic” (Best et al., 2017), the authors noticed that speech perception of the dynamic target condition was degraded, presumably because the target words appearing in a new unpredictable direction were brief, and the eyes could not reorient quickly enough to the new direction. Another study from the same laboratory, Roverud et al. (2018), came to the same conclusion with a slightly different paradigm. They measured the sensitivity of detection of the congruence between an acoustically presented word, among other competing words, and a word printed on a monitor screen (which also indicated the direction of the target). Favre-Félix et al. (2018) compared a gaze-steered simulated beamformer against nonsteered conditions when listening to three simultaneous spatially separated sentences such that the target sentence was clearly indicated by a visual cue. Gaze steering provided better recall of the target sentences than no-steering.

The idea of gaze-steering technology comes with the assumption that the gaze orientation is a better predictor of the listener’s attention than head orientation, especially in meetings and conversations (Vrzakova, Bednarik, Nakano, & Nihei, 2016). People often undershoot sound targets with their head angle (Grange & Culling, 2016 b), although head movement magnitudes vary between individuals (Fuller, 1992). However, people usually look at the faces of the participants in a conversation; gaze is directed at talkers much more than other (nonspeaking) participants (Coutrot & Guyader, 2014; Vertegaal, Slagter, van der Veer, & Nijholt, 2001). Lip reading can also help people to understand speech in noisy environments (MacLeod & Summerfield, 1987), and people with hearing loss report greater use of this strategy in conversations than normal hearing people (Monzani, Galeazzi, Genovese, Marrara, & Martini, 2008). Nevertheless, the aforementioned studies of gaze-controlled beamforming have yielded mixed results regarding whether such technology would be beneficial in terms of improved speech intelligibility.

A problem which has not been fully considered previously is that people in conversations move their heads, especially when they switch attention from one person to another. Participants in one study (Hart et al., 2009) were allowed to move their heads, and the authors noted that selection of the target using head angle alone was slower than eye selection. Head movements were restricted in the other three studies (Best et al., 2017; Favre-Félix et al., 2018; Roverud et al., 2018).

When listening with a highly directional beamformer, any orientation errors of the acoustic beam lead to attenuation of potentially relevant sounds (Archer-Boyd et al., 2018). On the one hand, greater off-axis attenuation of a (narrow) beam oriented toward the target will provide better suppression of background noise when the target is stationary, but it will also decrease the audibility of new, off-axis, targets. On the other hand, less off-axis attenuation (a wide beam) will decrease the effect of target source movements and beam-orientation errors, but it comes with the price of a decreased maximum attenuation of noise. In all four previous studies (Best et al., 2017; Favre-Félix et al., 2018; Hart et al., 2009; Roverud et al., 2018), the shape of the beam, which defines the magnitude of off-axis attenuation, was constant throughout the experiments.

To address the limitations noted earlier, this study aimed to directly compare speech intelligibility between listening with an eye gaze-controlled beamformer (GAZE) and listening with a head-controlled beamformer (HEAD) in a dynamic “cocktail party” situation, when a listener is free to move their head. The study also aimed to investigate how the individual listeners’ head-orientation strategies interact with the beamforming technology and beam width. The experiment was intended to simulate a conversation-like situation in a noisy environment in which participants have to switch attention from one place to another at irregular intervals although the simulation lacked natural conversation-related turn-taking cues; thus, the participants were not informed upfront about the upcoming target switch. The beam, in this study, was either highly directional (NARROW), moderately directional (WIDE), or omni-directional (OMNI). To enable direct comparison between the HEAD and the GAZE conditions, the effect of different beam widths on the overall noise level was canceled out. This was done by normalizing the target level in each beam width condition to yield speech intelligibility of 84.4% correct with the beam fixed at the target and the participant oriented toward the target. Therefore, to avoid confusion, the results are henceforth designated “normalized speech intelligibility” (NSI).

On the basis of the previous work reviewed earlier, and in the context of the experimental framework just described, we hypothesized as follows:

- With the target direction remaining static, gaze orientation would fixate targets more accurately than head orientation, and in consequence, GAZE would perform better than HEAD in terms of NSI.
- In the case of the target direction changing unpredictably, GAZE control would not provide higher NSI compared with HEAD control.
- The relative difference in performance between the GAZE and HEAD conditions would be dependent on the beam width and individual head-orientation strategy.

## Methods

### Listeners

**Figure 1.**
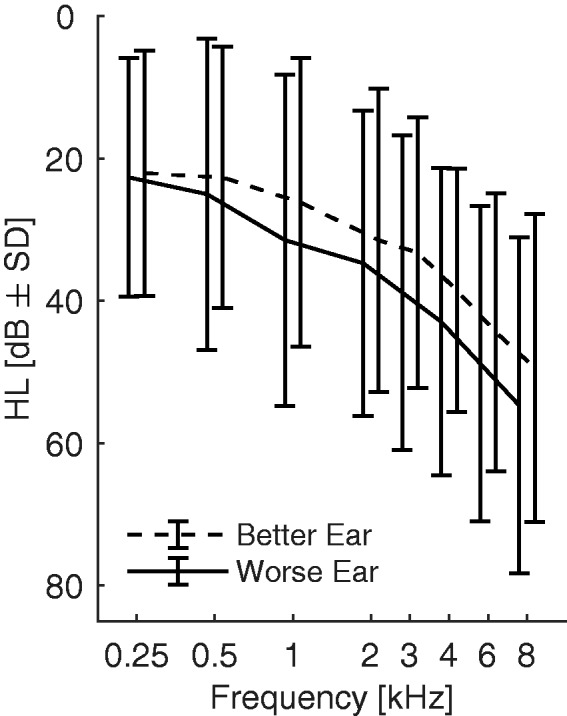
Average audiograms of better and worse ears. *SD* = standard deviation.

Participants (*n* = 17, seven women, mean age 64 years, standard deviation 11, age range 31–74 years) had a range of symmetrical hearing thresholds (Figure 1), with better ear four-frequency average (BEFFA; 500 Hz, 1 kHz, 2 kHz, and 4 kHz) of audiometric pure tone thresholds ranging from 5 to 55 dB HL. Symmetry was defined as a maximum of 15 dB HL difference of the four-frequency average between the ears. Participants with impaired hearing had mildly sloping hearing losses such that the slope of the four-frequency pure tone thresholds of each of the participants was less than 15 dB/octave. The participants were paid for participation, and all of them had previous experience with psychophysical experiments. All but one of the participants had English as their first language and were naive to the details of this study. The participant who did not have English as his first language (author L. H.) had similar data to other participants. The experimental protocol was approved by the School of Medicine Research Ethics Committee of the University of Nottingham (ref. no.: 38-1706). All participants provided informed consent.

### Environment

The experiment was conducted in a double-walled soundproof booth (IAC Acoustics, Winchester, UK). The room had internal dimensions 4.6 m × 4.1 m × 2.5 m (l × w × h), a carpeted floor, and walls and ceiling covered with sound-absorbing foam wedges (AFW305, Comfortex Acoustics, Oldham, UK). It had a door and one window. The reverberation time RT_60_ of a broadband signal was 0.086 s (measured with a 1/2″ free-field microphone, 40AE, GRAS Sound and Vibration, Holte, Denmark).

**Figure 2.**
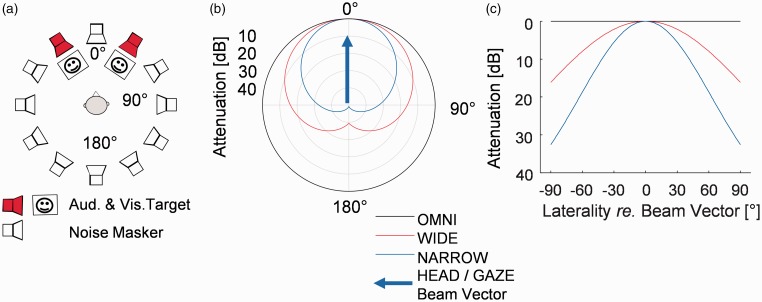
(a) Schematic of the experimental setup. Only one target sound and one face in the same azimuth were presented at a time. (b) Attenuation patterns of the simulated beamformers facing the midline in a polar coordinate system. (c) Attenuation patterns of the simulated beamformers in a Cartesian coordinate system. The beam vector was controlled either with the horizontal gaze or with the horizontal head orientation. NARROW = highly directional; WIDE = moderately directional; OMNI = omni-directional; GAZE = gaze-controlled; HEAD = head-controlled.

The participants were seated on a chair in the middle of a loudspeaker ring (radius 178 cm), with 12 loudspeakers (VX6, Tannoy, Coatbridge, UK), spaced at 30° intervals, and positioned on loudspeaker stands at the approximate height of the participant’s head. The signal output level of each loudspeaker was corrected by a coefficient to provide matched sound levels. The coefficients were computed in a calibration procedure in which the loudspeakers played sine sweeps of a known intensity, and these were recorded via the calibrated microphone placed in the center of the loudspeaker ring. The one-third-octave band frequency response of the loudspeakers at the listener position was within ±3.3 dB from the mean response across loudspeakers in the frequency range from 500 to 5000 Hz. The sound stimuli were delivered through a multichannel digital signal processor (A16 MK-II, Ferrofish, Linz am Rhein, Germany) whose outputs were fed to three 4-channel amplifiers (SLA4, ART Pro Audio, Niagara Falls, NY). The room was further equipped with two flat-screen visual displays (23″, EliteDisplay E231, Hewlett-Packard) vertically located at the two possible target directions on the left (−30°) and the right (30°) of subject midline (0°; Figure 2(a)), and these monitors were placed on wooden cabinets below the loudspeakers. A third monitor display (15″, ET1515L, ELO Touch Solutions) was positioned on the participant’s midline 1 m away from the participant and below the plane of the frontal loudspeaker. This was used to provide feedback about the performance of the participant after each block of trials to maintain motivation and concentration. An optical motion-tracking system (Bonita 10, Vicon, Oxford, UK) with eight room-mounted cameras captured the position and orientation of the participant’s head at 100 frames/s using five reflectors mounted on a “crown” worn by the participants. A wearable eye-tracking device mounted on a spectacles-like frame (Pupil Labs, Berlin, Germany) was used to monitor eye gaze at 60 frames/s. The eye-tracking software (Pupil Capture, v 0.7.9) ran on a separate Linux machine. The output of the eye tracker was sent over the local network to the control computer, which used a Max/MSP (Cycling 74, Walnut, CA) script for real-time soundfield generation. Java (Oracle) and MATLAB (Mathworks, Natick, MA) scripts were used to control the pace and timing of the experiment. A conservative estimate of the delay of the whole signal processing chain was 32 ms, which we judged to be sufficiently short to have negligible effect in the context of the stimuli and tasks of this experiment. This estimate is based on the detailed knowledge of the hardware and software environment, but the precise delay was not formally assessed.

Pilot testing suggested that the room lighting interacted with the cameras of the eye trackers, and therefore ambient lighting was turned off during the testing (but not during the level normalization procedure, which was conducted before the main experiment). However, five participants complained that testing without room lighting made them feel uncomfortable. The lights were turned on for their sessions to motivate these participants to complete the test. Turning the lights on helped them to maintain concentration and keep their eyes open, which was essential for the eye tracking. Although this might have added some variability to our data, we did not notice any differences between these two groups of participants. No participant was in complete darkness, as some light spilled into the test room from the control room, and from the monitor displays, where a white background was always illuminated.

### Stimuli

The target sounds were spoken digits from one to nine (except seven) and they were spoken by 11 native English speakers (four women) with Scottish, English, and American accents. The words were spoken with normal voice, and one recording was used for each combination of a digit and a speaker, making in total 88 tokens. The original recordings were postprocessed such that the stimuli were of equal duration (572 ms between onset and offset, defined as −25 dB re. mean level) and peak level using the commands “Lengthen” and “Scale” of software PRAAT (Boersma & Weenink, 2017).

The sound levels of the target stimuli were normalized individually for each participant and each beam pattern before the main experiment in order to achieve equal speech recognition performance (84.4% correct; Levitt, 1971) in a reference condition with a fixed target position. For this reference condition, the target stimuli were presented only from the left target loudspeaker and the orientation of the peak of the simulated beamformer was locked toward the target loudspeaker (i.e., there was no tracking of head or eye movements). During the measurement in the reference condition, the participants were instructed to orient their heads toward the left target, but the actual head orientation was not monitored. By normalizing each beamformer condition, we were able to isolate the interactions of movement and beam width and effectively remove the static effect of beam attenuation pattern. This level normalization was the only form of individualized compensation for hearing loss included in the setup.

During both the normalization procedure and the main experiment, target stimuli were presented together with continuous speech-shaped noise (International Telecommunication Union, 1988), such that uncorrelated noise samples were presented from all loudspeakers (Figure 2(a)) except the two which were used for presenting the targets.

On each presentation of the target during the main experiment, the target sounds were accompanied with a static picture of a face looking directly ahead on a white background (Boshcung, 2016). The face was presented on a monitor placed under the target loudspeaker (Figure 2(a)). It appeared simultaneously with the onset of the speech token and remained visible until the target changed direction.

The auditory stimuli were designed to simulate a conversation in a noisy environment. Thus, the target stimuli were presented every 1.5 s from one of the two possible target directions at ±30° (Figure 2(a)) in a pseudorandom sequence. The initial target direction was chosen at random, after which it switched with a pseudorandomly chosen interval of three, four, five, or six trials, such that there were 15 switches for each interval in each block within the experiment. A presentation of the target stimuli (sound + picture) was followed by a period for verbal response, which together formed a single trial. This gave 270 trials per block. The switch randomization was included because, in real conversations, the direction of new targets may not always be predicted.

### Beamformer Simulation

The motion- and eye-tracking data were used in combination with the loudspeaker setup to drive a real-time simulation of the acoustical processing which would be similar to a hearing aid system with a highly directional bilateral beamforming microphone array (Best et al., 2015). The principal differences between a hearing aid beamformer and our simulation were that our simulation fully preserved spatial properties of the sound, and the attenuation was independent of frequency. A real acoustic beamformer would degrade the spatial properties of the sound delivered to the ears, and the attenuation would vary with frequency. By using a frequency-independent beamformer, we aimed to provide an attenuation pattern that would be neutral with respect to the pattern provided by any specific implementation in a real beamforming microphone array.

This “simulated beamforming system” was the gaze- or head-direction-dependent attenuation pattern (Figure 2(b) and (c)) that was mapped onto the loudspeaker array. Henceforth, directional processing using the head-tracker data to determine focal axis is referred to as the HEAD control condition. Correspondingly, processing based on combined head and eye-tracking data is referred to as the GAZE control condition.

The output level of any given loudspeaker was attenuated according to one of the three possible directivity patterns, which could be NARROW, WIDE, or OMNI (Figure 2(b) and (c)). With the beam vector pointing directly toward a target loudspeaker, the overall noise attenuation (at the center of the array with the participant absent) provided by WIDE and NARROW was 8 and 12 dB respectively, relative to OMNI. The noise level in the OMNI condition, that is, when there was no effect of beamforming simulation, was 68 dB SPL. Note that the designation of NARROW and WIDE was for convenience only, even the WIDE pattern had a higher directional selectivity than a bilateral beamforming array which can be found in some hearing aids (Buechner et al., 2014).

### Procedures

Before the experiment, the experimenter explained the procedures to the participants and answered any questions. For each of the three beam width conditions, each participant then underwent the aforementioned speech intelligibility normalization procedure.

After the normalization procedure, the participant was fitted with the motion-tracking crown, the eye tracker, and a wearable microphone. First, the motion-tracking system was calibrated using special glasses with reflective markers that helped to exactly locate the tragi and the nose of the participant with respect to the motion-tracking crown. The calibration glasses were not worn during the subsequent testing. The eye tracker was calibrated using the automatic on-screen calibration provided by the eye-tracking software on another LCD flat-screen (40″, UE40ES5500, Samsung) that was moved 80 cm in front of the participant for this procedure only. It was removed for the actual testing.

All participants were asked to take off their glasses, to avoid interference with the eye-tracking device. However, one participant wore glasses during the experiment because he was not able to do the task without them, and in his case, it was possible to calibrate the eye tracker with the glasses on. All participants took part without wearing hearing aids.

For the main experiment, the task of the participant was to imagine that they were in a restaurant with two friends, one sitting on the left, one on the right, and that they were following a “conversation” between these two people. While listening to the “conversation,” the participant was instructed to repeat the presented digits out loud (to be picked up by the wearable microphone). If the participant could not understand completely or did not hear the target sound at all, they were asked to make their best guess or say a random digit. The experimenter was sitting outside the testing room and recorded the responses into a computer in real time. Responses to trial *n* which were recorded up to 100 ms after the offset of stimulus *n + *1 were assigned to trial *n*. The 100-ms cutoff was found based on a visual inspection of response-time histograms, and it reliably allocated responses to their correct trials.

The testing consisted of five blocks, each of which tested one of the five conditions. The first block was always the OMNI condition in which the simulated directionality was inactive. The subsequent four blocks had one of the four possible combinations of the two beam widths (WIDE and NARROW) and two beam control types (HEAD and GAZE). The order of the four conditions was randomly generated for each participant. The blocks were separated by short breaks during which the participants were encouraged to relax. After the experiment, the participants were debriefed.

### Analysis

All participants’ responses were analyzed to assess the performance by computing the number of correct responses and the percentage of correct responses for each experimental block. The latter values were then transformed into rationalized arcsine units (RAU; Studebaker, 1985) and used for the statistical analysis. The motion data—head and gaze horizontal angles—were analyzed in the periods when the target sound was on. All statistics, reported here, were computed using CLEAVE (Herron, 2005) and MATLAB software. Reported *p* values of the *F* statistics used in this text were corrected with the Greenhouse–Geisser epsilon to account for a likely violation of the sphericity assumption of the repeated-measures analysis of variance model. The data were also analyzed using linear mixed effect models as implemented in MATLAB (R2018b v9.5). The structure of the model was kept identical between the fixed and random effects using a diagonal covariance matrix for the random effects. The models were fit with maximum likelihood estimation with the default optimizer, and likelihood ratio tests were conducted to assess the difference between any one model and an alternative model.

## Results

**Figure 3.**
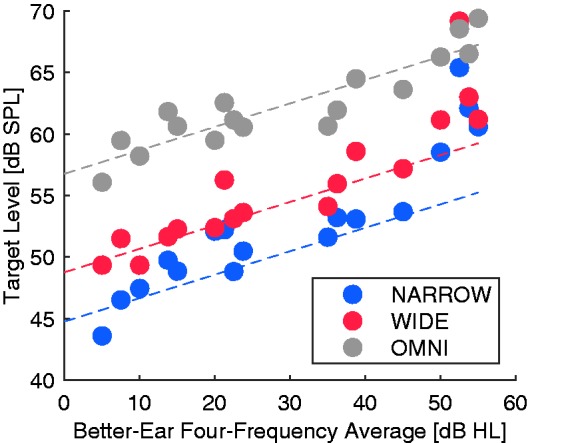
Target sound levels as a function of hearing status (measured as BEFFA) for each of the three beam width conditions (depicted by color) and for each individual (full dot). Gray dashed line is the best linear fit to the OMNI data, and red and blue dashed lines represent −8 dB and −12 dB, respectively, relative to the gray line. NARROW = highly directional; WIDE = moderately directional; OMNI = omni-directional.

Figure 3 shows the individual target sound levels resulting from the normalization procedure, as a function of participants’ hearing losses. To a large extent, the individual data points conform to a prediction that the received SNR for a given participant should remain constant for equal speech recognition performance across conditions (i.e., target level 8 and 12 dB lower for the WIDE and NARROW beams, respectively, relative to OMNI). However, the three participants with the greatest hearing losses appear to require somewhat elevated target sound levels, suggesting that for them, absolute audibility may have been a determining factor, as well as SNR.

In the main experiment, NSI performance was mainly determined by whether the target direction was static or switching. When the target direction was static (i.e., the previous trial had the same target direction as the current trial), the across-subject mean NSI performance was 79.3 ± 1.3 RAU computed across all conditions except OMNI. When the target direction switched, the performance dropped to 23.6 ± 3.8 RAU, *t*(16) = 14.93, *p* < .001. The drop of the performance in the beamforming conditions thus relates to the direction of the beam vector in the switching trials. Performance in the OMNI condition in the static trials was 89.5 ± 1.2 RAU, which was lower, *t*(16) = 3.53, *p* = .003, than the performance in the switching trials, 95.22 ± 1.85 RAU. The small difference in performance between the switching trials and the static trials in the OMNI condition may relate to the acoustic head-orientation benefit (Grange & Culling, 2016a) as participants could benefit from the acoustics if the ear (rather than the nose) was turned toward the target sound.

To identify whether the recovery period after a target switch (“time after switch”) was greater than 1.5 s, the NSI values of the static target direction trials were analyzed using analysis of variance with factors of beam width, beam control type, and time after switch (i.e., number of trials after switch of target location). The factor time after switch, *F*(4, 52) = 0.97, *p* = .42; the interactions between time after switch and beam width, *F*(4, 52) = 2.48, Geisser–Greenhouse ɛ = .51, *p* = .098; time after switch and beam control type, *F*(4, 52) = .73, *p* = .57; and time after switch, beam width, and beam control type, *F*(4, 52) = 1.32, *p* = .27, were all statistically insignificant. Therefore, it appears that recovery was complete by 1.5 s, and the temporal factor (time after switch) was disregarded for further analysis. The static target trials and switching target trials were then analyzed separately.

**Figure 4.**
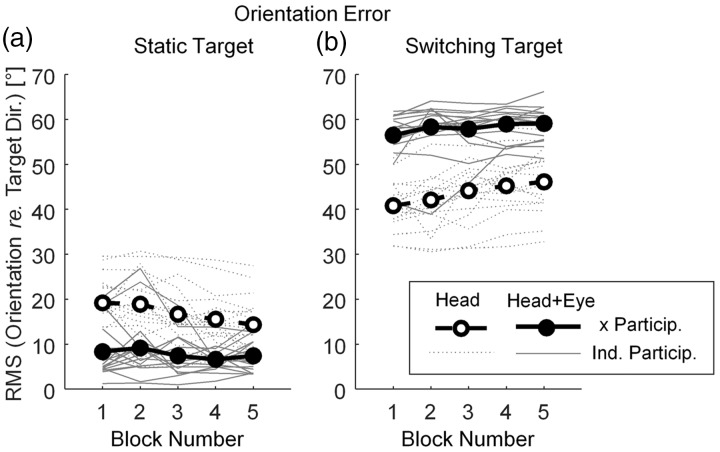
Absolute orientation errors computed as root-mean-square orientation error of the horizontal head orientation (open circles connected by dashed line segments) and horizontal gaze (head + eye) orientation (filled circles connected by solid line segments) over the course of the experiment. Each data point, for each participant, was obtained by averaging horizontal (head or gaze) angle over the duration of the target word and over the repetitions of given condition. Each connected series of dots with lines shows data of one participant. Data in the static target trials (a) and in the switching target trials (b) are shown.

Participants’ orientation strategies were characterized in terms of orientation errors with respect to the target direction. Figure 4 shows absolute orientation errors of individual participants as a function of experimental block number in static trials (Figure 4(a)) and switching target trials (Figure 4(b)). The orientation error was computed as root-mean-square head or gaze horizontal orientation with respect to target direction. The distribution of dotted lines in Figure 4 (for individual participants) indicates high across-participant variability of head-orientation errors, in both static and switching target trials. In the static trials, gaze-orientation errors (7.78° ± 0.35°) were significantly smaller, *t*(16) = 6.01, *p* < .001, than the head-orientation errors (16.93° ± 0.46°). This is in line with the hypothesis that people direct their gaze toward targets more accurately than their heads. While in the switching target trials, gaze-orientation errors (58.14° ± 0.24°) were significantly larger, *t*(16) = 10.79, *p* < .001, than head-orientation errors (43.67° ± 0.37°) indicating that participants could not reorient to the new location while the new word was presented.

To illustrate that the participants were following the targets within each block, Pearson’s correlation coefficients between the orientation, in static target trials, and the target direction were computed for each participant. The across-subject means of the coefficients were .96 and .99 for the head orientation and gaze (head + eye) orientation, respectively (the coefficients were *z*-transformed before the computation of the mean and the *z*-transformed means were transformed back).

To evaluate consistency of head-orientation strategy across conditions and across time (experimental blocks), the orientation errors computed for each block were analyzed using linear mixed effect models. OMNI data were excluded from the analysis because they were not subjected to across-block randomization. A model including the intercept and the factor of block number fitted data better than the intercept-only model, χ^2^(2) = 16.96, *p* < .001. This model suggests that head-orientation error decreased on average by 1.26° per block, *t*(52) = 4.36, *p* < .001. Alternative models which included intercept, block number, an additional factor, and interaction of block number and the additional factor were tested for these additional factors: beam control type, χ^2^(4) = 5.12, *p* = .27, beam width, χ^2^(2) = 6.05, *p* = .19, interaction of beam width and control type, χ^2^(2) = 5.34, *p* = .25, age (for this comparison we fit the model with a full covariance matrix), χ^2^(9) = 10.78, *p* = .29, and BEFFA, χ^2^(4) = 11.43, *p* = 1. None of these alternative models improved the fit of the original model. In other words, beam width, beam control type, and age did not affect the patterns of head movement.

**Figure 5.**
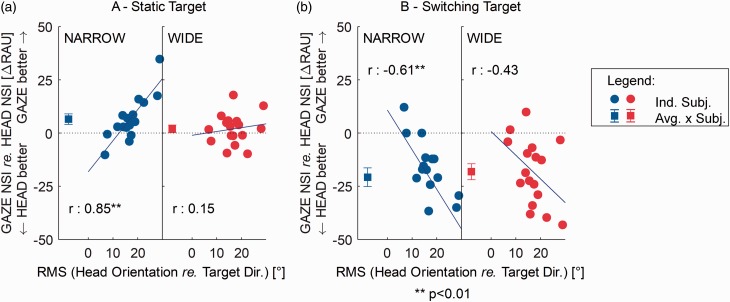
Relative GAZE versus HEAD NSI performance for all individuals and conditions. (a) Static target direction. (b) Switching target direction. Both panels (a and b) show NSI performance in the GAZE condition, relative to that in the HEAD condition, for the NARROW (left subpanels) and WIDE (right subpanels) conditions. Positive values indicate GAZE performance better than HEAD performance. The circle symbol for each individual participant is located on the *x*-axis according to their overall RMS head-orientation error. Square symbols in all panels show group means. Error bars show standard errors of the mean. The *r* values are Pearson’s correlation coefficients. Sloping blue solid lines show best fitting regression lines. NARROW = highly directional; WIDE = moderately directional; OMNI = omni-directional; GAZE = gaze-controlled; HEAD = head-controlled; RMS = root-mean-square; NSI = normalized speech intelligibility; RAU = rationalized arcsine units.

To directly compare GAZE and HEAD NSI performance in relation to individual propensity to head movements, Figure 5 shows the differential NSI performance of the two beam control types and two beam widths as a function of head-orientation error for each individual (circles) together with group means (squares). The head-orientation errors were averaged across blocks because the effect of block number was small relative to the across-participant variability and also because there was no effect of beam width or beam control type on head-orientation error.

In the static trials (Figure 5(a)), average NARROW GAZE performance was significantly higher than average NARROW HEAD performance, *t*(16) = 2.66, *p* = .016, (i.e., GAZE was better), but average WIDE GAZE was not significantly higher than average WIDE HEAD, *t*(16) = 1.13, *p* = .27. The correlation between head-orientation error and the NSI performance was high and significant for the NARROW beam (*r* = .85, *p* < .001). The correlation for the WIDE beam (*r* = .15, *p* = .57) was insignificant. Participants whose strategy was always to orient the head in between the targets (i.e., low propensity to head movements and high-orientation error) benefited from the NARROW GAZE relative to NARROW HEAD more than participants whose strategy was always to orient the head toward the target. However, most people were in between the two extremes. The difference between GAZE and HEAD increased in proportion to the amount by which the head was undershooting the target direction (and gaze direction). Furthermore, the correlation decreased for the WIDE beam because the wider beam also reduced the effect of motion-related errors on performance.

In the trials when the target direction switched unpredictably (Figure 5(b)), average NSI performance with HEAD control was significantly better than with GAZE control in both NARROW, *t*(16) = 4.73, *p* = .001, and WIDE, *t*(13) = 4.85, *p* < .001, conditions. The correlation between NSI performance with GAZE versus HEAD control and head-orientation error was significant in the NARROW condition (*r* = − .61, *p* = .01) and insignificant in the WIDE condition (*r* = −.43, *p* = .089). Performance in the switch trials was mainly determined by the fact that at the time of presentation of the new target, participants were still focusing on the previous target. As head orientation tends to systematically undershoot a static target, orientating to the wrong target is “less wrong” for the head than for the eyes. Hence, when the target switches location, the head-controlled beamformer will attenuate the new target direction slightly less than the gaze-controlled beamformer. Furthermore, the larger the head-orientation error is during the static condition, the stronger this effect will be. This leads to a negative correlation between the relative NSI performance of the HEAD and GAZE conditions and head-orientation error. The correlation was weakened in the WIDE condition as more participants were then likely to hear out the new target due to the wider beam.

### Confounding Factors

Here, we assess the contribution of possible confounding factors of age, hearing status, and block number on NSI performance. First, NSI data of static trials for each block (except the first block with the OMNI condition) were fitted using a linear mixed effect model with factors of beam width, beam control type, and head-orientation error in each block. These were the main factors in the previous analysis. This model fitted the data significantly better than the intercept-only model, χ^2^(14) = 28.27, *p* = .013, and all of its main effects, intercept: *t*(60) = 5.85, *p* < .001; beam control type: *t*(60) = 2.34, *p* = .022; beam width: *t*(60) = 3.02, *p* = .004; head-orientation error: *t*(60) = 2.32, *p* = .023, and all of its interactions, Beam Control Type × Beam Width *t*(60) = 2.03, *p* = .047; Beam Control Type × Head-Orientation Error: *t*(60) = 3.54, *p* < .001; Beam Width × Head-Orientation Error: *t*(60) = 2.46, *p* = .017; Beam Control Type × Beam Width × Head-Orientation Error: *t*(60) =2.88, *p* = .005, were significant.

In the next step, for each of these confounding factors, the aforementioned model was extended with the confounding factor and all interactions of this new factor. None of these factors significantly improved the model: the factor of age, χ^2^(16) = 33.30, *p* = 1, BEFFA, χ^2^(16) = 33.08, *p* = 1, and block number, χ^2^(16) = 13.05, *p* = 1.

## Discussion

Highly directional beamformers improve speech intelligibility when the target sound is static and when the beam is fixed to the target direction. However, in daily conversations with multiple people, the listener with head-fixed beamformers may miss the initial portion of the speech from a new unexpected direction. This study investigated listening to a simulated two-talker conversation with both head-controlled and gaze-controlled beamforming involving two beam widths. The results supported the hypothesis that gaze followed the target more accurately than head, but only when the target was static. When the target changed direction without warning, the gaze orientation was usually highly imprecise. These behavioral patterns were then reflected in the speech intelligibility performance and the results can be summarized as follows: (a) on average, gaze-controlled beamforming, in comparison with head-controlled beamforming, may improve speech intelligibility of the target speaker in a two-person conversation, if the off-axis target speaker location is static, and if the beam is sufficiently narrow for a given spatial separation of the targets (60° in this experiment); (b) on average, head-controlled beamforming, in comparison with gaze-controlled beamforming, may provide higher speech intelligibility when the target unpredictably switches back to a previous direction (i.e., the moment of turn-taking); (c) widening the beam decreases the difference between the two beam control types; (d) the relative difference between gaze-controlled and head-controlled beamforming in a two-talker scenario further depends on individual head-orientation error, which varied between participants to a high degree (only participants whose head-turning significantly undershot the target direction could benefit from gaze steering of the acoustic beam); and (e) listening with a highly directional acoustic beamformer (either gaze-controlled or head-controlled) always incurs a speech intelligibility penalty related to orientation behavior, which will be pronounced for narrower beams and when the target direction changes. This performance decrement might be compensated by the SNR benefit of the beam, but the trade-off between the signal-to-noise improvement and orientation-related speech-intelligibility decrement remains to be investigated. The current experimental design did not permit the assessment of the overall benefit of different beams and control types because performance level was normalized for each of the beam width conditions.

We were also interested to see whether participants adapted their head-orientation strategy in response to the experimental conditions. Although the participants were not informed about the details of signal processing, the results suggested that the participants tended to orient their head slightly more toward the target as the experiment proceeded, which could be an indication that people adapted to the experimental situation. However, this was independent of the beam width, beam control type, age, and hearing loss. The participants either learned some features of the experimental design, which helped them to do the task more efficiently, or this trend of increasing movement is a sign of fatigue (which seems counterintuitive). Other than this, our data do not provide evidence to support any specific hypotheses regarding what caused the orientation strategy adopted by participants, although the literature does suggest that individuals show different traits of propensity to head movements (Fuller, 1992).

Overall, the present results support the conclusions of previous studies (Best et al., 2017; Roverud et al., 2018) that eye gaze cannot reorient quickly enough to focus on brief utterances from new and unexpected target locations. Our data suggest that the reorientation does not influence speech performance after 1.5 s, which is consistent with the report of the previous study (Roverud et al., 2018), although the stimuli were not presented at the same time intervals. This result is also consistent with a 500- to 750-ms average gaze transition time over 60° (Best et al., 2017), and the transition times of head movements toward audiovisual targets at similar eccentricities as in this study, which usually have been completed after 1.5 s (Zambarbieri, Schmid, Versino, & Beltrami, 1997). Furthermore, this study found an NSI benefit of the GAZE condition for static lateral targets, which is consistent with all previous studies, which used targets at lateral and frontal positions (Best et al., 2017; Favre-Félix et al., 2018; Roverud et al., 2018).

### Limitations of the Study

The current design aimed to simulate a conversation-like situation in a noisy environment with multiple participants. Although the design captured some aspects of such situations, other aspects were limited. The participants saw only a static picture at two lateral directions and loudspeakers, whereas real conversations take place in spaces which are much richer in terms of content and context. The auditory stimuli in this experiment were single words from a closed set presented every 1.5 s, which allowed the participants to give an immediate and precise response, whereas in a real conversation, diverse acoustic and nonacoustic cues can help people to predict turn-taking between talkers engaged in a conversation (Holler, Kendrick, Casillas, & Levinson, 2016). Speech prosody, semantic cues, gestures, facial expressions, gaze of the talkers, micropauses, social context, and other cues form a very complex interplay that facilitate communication and understanding of what is being said. This gives a lot of cues for prediction for the conversational turn and people can look at the target well before they move their head toward the new target, but in this experiment the turns were always unpredictable. Therefore, it was not possible to observe the benefit when the person has a temporal cue at the timescale 0 to 1,500 ms when it is most likely to observe the GAZE versus HEAD benefit.

Moreover, the participants were instructed to look at the target and repeat the digit. However, in real conversations, people do not always look at the speakers (Vertegaal et al., 2001), for instance, when speaking, or during the moments of composing a reply. Thus, although the task itself was demanding, there was only a limited engagement of high-level and integrative cognitive processes in the task.

The beam widths used in this study, both NARROW and WIDE, were more directional than directional microphones of most up-to-date hearing aids, which are closer to the OMNI condition (Dillon, 2012). However, some hearing aids are equipped with superdirectional bilateral beamformers, which can be as directional as our NARROW and WIDE conditions (Best et al., 2015; Buechner et al., 2014). The design focused on the effects of behavior, and therefore it also aimed to avoid the interaction between the low-frequency cutoff of real acoustic beamforming arrays and the level of hearing impairment of the individual (Best et al., 2017; Roverud et al., 2018). However, future studies should further investigate the extent to which high-frequency hearing loss interacts with the frequency response of microphone beamforming array in realistic situations.

Normalizing speech intelligibility performance prevented participants from experiencing the SNR benefit as it would be with a real device, and they did not have chance to learn the features of such a system and adapt their behavior. They were not informed about the details of system operation. It is likely that they did not try to exploit the system as much as possible. All these factors are crucial for understanding the benefits in real situations, and they should be addressed in future studies.

The population in the study was purposely selected to encompass a range of hearing statuses from normal hearing to moderate hearing loss. Beyond the level normalization at the beginning of the experiment, there was no frequency-dependent correction applied to account for the high-frequency hearing loss of some of the participants. However, all analyzed participants had relatively flat audiograms, so level-dependent changes in audible bandwidth were unlikely to confound the results (Boothroyd, 2008). Moreover, the factor of hearing status was not found to be a significant modifier of the statistical model of the data, despite a 50 dB HL range of BEFFA.

Although these limitations constrain the applicability of the results, we believe that this study provides valuable insights into the general ability of speech perception of spatially distributed targets under directional microphone processing. In future studies, some of these limitations might be overcome by employing free-listening tasks or active conversation in an immersive virtual reality system (Hendrikse, Llorach, Grimm, & Hohmann, 2018; Seeber & Clapp, 2017), which may lead to more natural behavior. Further improvement of the design could be achieved by employing simulations of hearing devices, with or without noise reduction schemes, to match the actual hearing impairment of an individual (Kayser, Herzke, Loshaj, Grimm, & Hohmann, 2017).

## Conclusion

This experiment investigated speech intelligibility performance in a two-talker scenario with a 60° separation in spatially distributed continuous speech-shaped noise. The participants followed the targets with gaze orientation more accurately than with head orientation, but their orientation was accurate only when the target was static. Gaze could not reorient quickly enough to the unpredictably switching target and therefore gaze orientation had large errors in these instances. Head orientation had lower error when the target was changing location. These patterns drove the relative speech intelligibility between the two beam control type conditions. The results were further influenced by the beam width and individual propensity to head movements.

The overall speech intelligibility benefit of the proposed beam control types in real situations remains to be established. The absolute performance will be a trade-off between large SNR improvements due to beamforming, when listening to a static target, at the expense of a decrease in SNR during turn-taking. In real conversations, turn-taking is rarely completely unpredictable, therefore people may reorient toward the next speaker before the onset of the new utterance. Thus, GAZE control has a good potential to preserve high-SNR benefits even when targets change location.

Future research may focus on technologies that preserve the benefit and minimize the adverse effects of switching. Such technologies might work by analyzing listening attention (O’Sullivan et al., 2014) and building models of the conversational scenes either based on the behavioral or acoustical information. Device technologies can exploit user behavior, but they can also influence behavior, therefore the effects of such technologies on behavior ought also to be studied. Future research may be conducted with a wider range of hearing-impaired participants, with real devices, and real conversation partners.
